# Glutamate plasticity woven through the progression to alcohol use disorder: a multi-circuit perspective

**DOI:** 10.12688/f1000research.9609.1

**Published:** 2017-03-21

**Authors:** Lara Hwa, Joyce Besheer, Thomas Kash

**Affiliations:** 1 Department of Pharmacology, University of North Carolina School of Medicine, Bowles Center for Alcohol Studies, Chapel Hill, NC, 27599, USA; 2 Department of Psychiatry, University of North Carolina School of Medicine, Bowles Center for Alcohol Studies, Chapel Hill, NC, 27599, USA

**Keywords:** glutamate, alcohol, addiction, two-bottle choice, self-administration, drinking in the dark, intermittent access to alcohol, chronic intermittent ethanol vapor

## Abstract

Glutamate signaling in the brain is one of the most studied targets in the alcohol research field. Here, we report the current understanding of how the excitatory neurotransmitter glutamate, its receptors, and its transporters are involved in low, episodic, and heavy alcohol use. Specific animal behavior protocols can be used to assess these different drinking levels, including two-bottle choice, operant self-administration, drinking in the dark, the alcohol deprivation effect, intermittent access to alcohol, and chronic intermittent ethanol vapor inhalation. Importantly, these methods are not limited to a specific category, since they can be interchanged to assess different states in the development from low to heavy drinking. We encourage a circuit-based perspective beyond the classic mesolimbic-centric view, as multiple structures are dynamically engaged during the transition from positive- to negative-related reinforcement to drive alcohol drinking. During this shift from lower-level alcohol drinking to heavy alcohol use, there appears to be a shift from metabotropic glutamate receptor-dependent behaviors to N-methyl-D-aspartate receptor-related processes. Despite high efficacy of the glutamate-related pharmaceutical acamprosate in animal models of drinking, it is ineffective as treatment in the clinic. Therefore, research needs to focus on other promising glutamatergic compounds to reduce heavy drinking or mediate withdrawal symptoms or both.

## Introduction

Glutamate, the most prevalent excitatory neurotransmitter in the central nervous system, has long been associated with the excitotoxicity of alcohol withdrawal. Repeated episodes of alcohol withdrawal can generate aberrant behaviors such as hypermotility and increased seizures, which are classically thought to be related to an excitable state caused by increased glutamate action in the brain ^1– 4^. These hyperglutamatergic periods of alcohol deprivation between heavy drinking events may be kindled across time, in a process like electrophysiological kindling ^5^. Since this hypothesis is generally well accepted in the field, many have explored glutamatergic targets for new alcohol use disorder medications ^6^. However, since an acute injection of ethanol also increases glutamate in the nucleus accumbens (NAc) ^7^, a site heavily associated with both reward and stress, it suggests that there is a continuum of engagement through the transition from low to heavy drinking regulated by glutamate signaling. We focus on circuits that become recruited among subcortical structures beyond the classic mesolimbic-centric perspective.

There are distinct pharmacological classes of glutamate receptors, including ionotropic (iGluR) and metabotropic (mGluR) glutamate receptors and glutamate transporters that have been linked to a wide variety of alcohol-related phenotypes. In brief, iGluRs encompass α-amino-3-hydroxy-5-methyl-4-isoxazolepropionic acid (AMPA) receptors with 1–4 subunits (GluA1–4), N-methyl-D-aspartate (NMDA) receptors with two obligatory GluN1 subunits and combinations of GluN2(A–D) assemblies, and kainite receptors (GluK1–5). GluN receptors are more sensitive to alcohol than GluA and GluK ^8– 10^. Also, allosteric modulation of the GluN2B binding site can produce changes in alcohol-related behaviors. In contrast to the ligand-gated cation-selective ion channel iGluRs, mGluRs are G-protein-coupled and form three distinct classes: group I (mGluR1 and mGluR5), group II (mGluR2 and mGluR3), and group III (mGluR4, mGluR6, mGluR7, and mGluR8). Glutamate clearance in the synapse can be controlled by reuptake through transporters like excitatory amino acid transporters (EAATs) and adenosine transporters (equilibrative nucleoside transporter, or ENT) into glia and vesicular glutamate transporters into neurons. This review synthesizes the extant behavioral pharmacological findings for the role of glutamate, its receptors, and its circuitry throughout the brain in several stages of the transition to alcohol use disorder. In light of clinical literature, three general phases within alcohol use disorders are discussed: low-level drinking, binge drinking, and heavy drinking with withdrawal. We highlight specific animal behavior protocols in these three categories, but importantly these methods can be applied among all phases in the development of alcohol dependence.

## Low-level drinking

Ethanol consumption that causes less than 0.08 g/dL (or 80 mg/dL) blood alcohol concentrations (BACs), less than 17 mM in the brain, is considered a low dose. Typical low-alcohol doses would be equivalent to a social drinker with BACs in the range of 0.015–0.025 g/dL (15–25 mg/dL). However, a BAC of 0.04 g/dL is classified as driving under the influence (DUI) for commercial drivers or previous DUI offenders ( http://www.dmv.org/). With rodents that can readily metabolize alcohol, higher gram per kilogram (g/kg) alcohol concentrations may lead to BACs under 80 mg/dL.

Acute sub-intoxication doses of alcohol ingestion in humans can cause reduced strength of evoked field potentials in the prefrontal cortex (PFC), suggesting reduced excitability and functional connections ^11^. This is concordant with a 0.375 g/kg ethanol injection inhibiting PFC firing rate by approximately 20% versus baseline in anesthetized rats ^12^. In general, there is a paucity of clinical data for low-level alcohol consumption and glutamate activity because low-level drinkers are compared with heavy drinkers instead of abstinent people in clinical research.

### Two-bottle choice

Two-bottle choice (2BC) involves offering the option to drink either a diluted ethanol-containing solution (concentrations range from 3 to 30%) or water for a fixed amount of time ( Table 1). 2BC allows for the measurement of both voluntary consumption and ethanol preference over water and can be used as a single protocol or be combined with others to generate the desired level of drinking. In other words, first-day BACs may indicate low-level drinking, but weeks of 2BC could produce intoxicating BACs. This section focuses on 2BC studies that assess baseline ethanol preference, but daily limited-access studies that generate more binge-like drinking are discussed in the next section.

**Table 1.** Descriptions of alcohol-related protocols.

Method	Details	Ethanol g/kg achieved	Key references
Two-bottle choice (2BC)	3–20% ethanol given in one bottle with a secondary bottle of water usually for 24 hours	≤10 g/kg per 24 hours (mice)	McClearn and Rodgers ^183^; Belknap, Crabbe, and Young ^184^
Operant self-administration	9–15% ethanol (some add 2% sucrose) reinforcements are self-administered with cue for 30–60 minutes	≤90 mg/dL BAC (rats), ≤200 mg/dL BAC (mice); ≤1.5 g/kg per 30 minutes (rats), ≤3 g/kg per 1 hour (mice)	Elmer, Meisch, and George ^185^; Melendez *et al*. ^186^; Faccidomo *et al*. ^57^
Cue-induced or stress- induced reinstatement	10–15% ethanol reinforcements are self- administered with cue, then extinction after cue is no longer paired with delivery of ethanol, finally reinstatement of ethanol-seeking behavior (lever pressing) occurs after ethanol-related cue or stressor is given	≤90 mg/dL BAC (rats), ≤200 mg/dL BAC (mice); ≤1.5 g/kg per 30 minutes (rats), ≤3 g/kg per 1 hour (mice)	Lê *et al*. ^187^; Chaudhri *et al*. ^188^; Shaham *et al*. ^46^
Alcohol discrimination	Sucrose or food pellet reinforcement given upon pressing the correct response lever after ethanol or vehicle injection; test sessions involve injecting a novel drug and measuring lever selection	0.5–2 g/kg injection, intraperitoneally or *per os*	Grant ^64^; Kostowski and Bienkowski ^65^
Drinking in the dark	3 hours into the dark photoperiod, one bottle of 20% ethanol is given for 2–4 hours (mice)	≤3 g/kg per 2 hours; ≤7 g/kg per 4 hours (mice)	Rhodes *et al*. ^90^; Thiele, Crabbe, Boehm ^91^
Scheduled high alcohol consumption	Water restriction for all but 90 minutes–10 hours, and every 3rd/4th day 5, 7, or 10% ethanol is substituted for 10–30 minutes followed by water	≤2 g/kg per 30 minutes; ≤100 mg/dL BAC (mice)	Finn *et al*. ^104^; Tanchuk *et al*. ^44^
Multiple scheduled access	Four 1-hour access periods to 15% and 30% ethanol separated by 2 hours starting 1 hour into dark cycle, 5 consecutive days/week	≤2 g/kg per 1 hour; ≤130 mg/dL BAC (rats)	Murphy *et al*. ^106^; Bell *et al*. ^107^; McBride *et al*. ^109^
Alcohol deprivation effect	5–20% ethanol given every day for 6–8 weeks with a 3- to 6-day deprivation then resumption of drinking	Additional 2 g/kg per 24 hours over baseline (rats); additional 4 g/kg per 24 hours over baseline (mice)	Sinclair *et al*. ^189^; Spanagel *et al*. ^112^; Melendez *et al*. ^190^
Intermittent access to alcohol	Every other day, 2BC of 20% ethanol and water is given for 24 hours, repeated for 4+ weeks	≤250 mg/dL BAC (rats), ≤200 mg/dL BAC (mice); ≤10 g/kg per 24 hours (rats); ≤25 g/kg per 24 hours (mice)	Wise ^131^; Simms *et al*. ^132^; Hwa *et al*. ^135^; Carnicella, Ron, and Barak ^133^
Chronic intermittent ethanol vapor	14-hour ethanol vapor and 10-hour air (rats) or 16-hour/8-hour (mice), repeated for 4+ weeks	150–250 mg/dL BAC during exposure; post-exposure, 6 g/kg per 24 hours (rats); ≤4 g/kg per 2 hours (mice)	Goldstein *et al*. ^191^; O’Dell *et al*. ^192^; Lopez and Becker ^193^

Listed are popular animal protocols for alcohol drinking and the amount of alcohol given to the animal. These methods produce relevant blood alcohol concentrations (BACs) in rodents and are not restricted to a low, episodic, or heavy drinking category. Protocols can be repeated to generate the intended level of drinking or be combined for more exploration of the drinking behavior.

The studies are unequivocal that NMDA and AMPA regulate 2BC drinking, and both competitive and non-competitive GluN antagonists reduce 2BC intake ^13– 15^. For example, NMDA and AMPA infused into the lateral hypothalamus can both increase 2BC consumption ^14^. However, GluN antagonists and glycine B site blockade can importantly reduce motor coordination to achieve these effects ^13– 16^. Similarly, GluN2A knockout mice show alcohol-induced impairments in motor coordination from wild-types (WTs) but do not show differences in consumption ^17^. Other glutamate-related knockout lines also do not differ in 2BC drinking compared with WTs (for example, AMPA GluR1, GluN1 glycine, and mGluR5) ^18– 21^. Pharmacological manipulations of mGluRs, specifically mGluR5 antagonists and mGluR7 agonists, are effective at reducing 2BC intake in rats ^22– 24^. In complementary experiments, blockade with mGluR7 antagonist MMPIP or shRNA in the NAc can increase low-dose alcohol intake and preference ^24, 25^. Since Homer2 knockout mice drink less than WTs in 2BC ^26^, it suggests that downstream signaling molecules are also important beyond glutamate receptor binding and clearance. It is worth mentioning that the US Food and Drug Administration (FDA)-approved medication for alcohol dependence, acamprosate, for which the glutamatergic mechanism of action is controversial, reduces 2BC drinking in rats ^27^. There is a glaring gap in the literature for which glutamatergic circuits in the brain may govern low-dose ethanol drinking. We need this critical information for insight into higher-dose plasticity.

Another important variable on the outcome of 2BC drinking and potential neuroadaptations is strain. Classic comparisons contrast between drinking behavior of C57BL/6J mice and DBA/2J mice, yet many inbred strains have been assessed for 2BC ^28^. Although specific sucrose-fading procedures can be used to induce ethanol drinking in DBA/2J mice (for example, 29) or bypassing ethanol taste altogether (for example, 30), this mouse strain drinks much less than C57BL/6J mice. 2BC preference may be related to strain differences in the effect of glutamate and NMDA on the brain *in vitro* ^31, 32^ and differences in gene expression in response to acute ethanol ^33^. Also, alcohol-preferring (P) rats, genetically selected for high alcohol drinking, have a loss of the mGluR2 receptors that may contribute to escalated alcohol intake ^34^. Comparing high-drinking and low-drinking strains caused from trait selection or from inbred lines would increase the understanding of how glutamate-related genes influence drinking behavior.

### Operant self-administration of alcohol

Operant self-administration is a powerful method for mice, rats, and monkeys to assess ethanol reinforcement. Via these methods, rodents will typically self-administer amounts ranging from 0.5 to 2 g/kg depending on factors such as session length, reinforcement schedule, and alcohol concentration by pressing a lever, spinning a wheel, or poking the nose into a receptacle ( Table 1). Uncompetitive GluN antagonists ketamine and memantine reduce operant responding for ethanol with mechanistic target of rapamycin signaling, likely regulating the anti-alcohol effects of ketamine ^35^. Both mGluR5 and mGluR1 blockade and mGluR7-positive allosteric modulation decrease alcohol self-administration in rats and mice ^36– 40^, particularly in the NAc ^41, 42^. As in 2BC studies, some have seen ethanol-induced sedation and hypnosis with mGluR5 antagonist MPEP and mGluR2/3 antagonist LY341495 ^43^ and non-specific reductions in sucrose self-administration ^39, 44^. This may be due in part to mGluR5 influencing D1 receptors in seeking behavior ^45^.

Self-administration training techniques are also useful to investigate cue-induced reinstatement, or seeking behavior, following extinction of the alcohol-paired cues ( Table 1). In operant self-administration protocols, cue-induced reinstatement or stress-induced reinstatement of alcohol seeking after a period of extinction training is also interpreted to be a form of relapse ^46^ ( Table 1). We discuss the literature here instead of the relapse section, as no alcohol is consumed during reinstatement tests. There have been mixed reports for the ability of competitive GluN antagonists to affect reinstatement ^47, 48^. For mGluRs, it is not surprising that mGluR5 antagonism and mGluR2/3 agonism reduce cue-induced reinstatement, alcohol seeking in Pavlovian spontaneous recovery, and enhanced sensitivity to the attenuation of conditioned reinstatement ^49– 52^, but there are varying reports for whether these compounds affect baseline self-administration. Gass *et al*. ^53^ found evidence for increased glutamate transmission from the basolateral amygdala (BLA) to NAc core during cue-induced reinstatement of alcohol seeking. Glutamate transmission and transport may be mediated through adenosine ENT1 ^54^ since N-acetylcysteine and ceftriaxone, which alter glial reuptake and release of glutamate, also alter alcohol self-administration ^55^. Downstream signaling molecules such as PKCε, ERK, and CaMKII/AMPA in the PFC and amygdala have been well established in alcohol self-administration and cue-induced reinstatement ^56– 59^. Specifically, amygdalar CaMKII/AMPA activation promotes self-administration and drinking ^60– 62^, whereas inhibition of CaMKII in the PFC increases the positive reinforcing effects of alcohol ^63^. Others have explored the activation of mGluR2 amygdala to hippocampus pathway in cue-induced alcohol seeking, where mGluR-mediated synaptic depression is impaired in the hippocampus ^34^. It seems that subregions of the amygdala and also the PFC are recruited during this low-level drinking.

### Alcohol-discriminative stimulus effects

Alcohol discrimination tasks are useful to assess the neurobiological mechanisms underlying the discriminative stimulus effects (for example, interoceptive effects) of low and high alcohol doses ( Table 1). However, it is important to note that these tasks do not involve alcohol drinking but rather experimenter-administered alcohol. We have known for decades that the discriminative stimulus properties of ethanol are mediated by GluNs and GABAA ^64– 66^. Specifically, lower alcohol doses (for example, 0.5–1 g/kg) engage GABA receptor systems whereas higher doses (>2 g/kg) involve NMDA receptor systems. Rats and cynomolgus monkeys can discriminate alcohol from glutamate release inhibitors and NMDA ligands, showing that they have partial alcohol-like effects ^67, 68^. This is different from the discrimination of acamprosate, where acamprosate fails to substitute for an alcohol cue, suggesting that it is not a substitution drug ^69^. Besheer *et al*. have shown that alcohol discrimination is co-regulated by mGluR5 in the NAc and the mGluR2/3 in the amygdala ^70– 73^ and that inhibition of MEK/ERK(1/2) in the amygdala, but not NAc, potentiates the effects of a low alcohol dose ^74^. Recent work with stress hormone corticosterone links both mGluR5 and mGluR2/3 in the sensitivity to alcohol ^75, 76^, suggesting a role for neuropeptide modulation of glutamatergic circuits. Furthermore, in addition to the NAc, a functional role for the medial PFC (mPFC) in modulating sensitivity to low alcohol doses has been shown ^66, 77^. An interesting contribution from the Holmes lab shows that GluN2B in corticostriatal circuits governs choice learning and choice shifting ^78^. Although this learning is not in the presence of alcohol, they show a dissociation between OFC GluN2B in choice shifting and dorsal striatum GluN2B in choice learning. These findings suggest it is possible that learning about alcohol through discrimination tasks recruits distinct populations of both iGluR and mGluR in subcortical sites, although more research is required to confirm how this contrasts from habitual learning in the striatum.

Overall, there is ample evidence demonstrating PFC plasticity in alcohol-seeking behavior and low-dose alcohol drinking at a stage engaging positive reinforcement and the euphoric effects of the drug. Although 2BC studies have tested several facets of the glutamate system using knockout mice, there is a gap of knowledge in iGluRs in alcohol self-administration studies. This may be confounded by the fact that competitive GluRN antagonists mimic the interoceptive properties of alcohol. More recent studies have implicated GluRA in the rostromedial tegmental nucleus in alcohol seeking ^79^. Another behavioral outcome of low-dose acute, self-administered alcohol (1 g/kg) is an increase in inter-male aggression in a subset of mice ^80^. Memantine, neramexane, and mGluR5 antagonist MTEP interacted with alcohol to further increase alcohol-heightened aggression in mice, whereas mGluR2/3 agonist LY379268 did not ^81^. CRF type-1 receptors regulate serotonin function from the dorsal raphe nuclei (DRN)- mPFC to alter alcohol-heightened aggression ^82^, so glutamate may influence the mPFC for the expression of low-dose alcohol-related behavior.

## Episodic drinking through binges and relapse

Binge drinking, defined as BACs greater than 0.08 g/dL or 80 mg/dL within 2 hours, is common among most strata of US adults and leads to an increased susceptibility for developing chronic alcoholism ^83, 84^. This section focuses on hazardous, episodic, binge drinking. However, epidemiological reports have found that there are almost as many binge-drinking episodes among moderate drinkers as among heavy drinkers in the US ^83^, so binge and relapse behavior represents the hazardous transition between moderate and heavy drinking. We focus on changes in glutamate plasticity to inform us on dramatic neurobiological events across species.

Binge alcohol drinkers have increased glutamate-to-creatine ratios and lower GABA concentrations in the anterior cingulate cortex (ACC) than do low alcohol drinkers ^85, 86^ presumably with glutamatergic perturbations. Repeated 2–3.4 g/kg alcohol injections increase accumbal and hippocampal glutamate compared with water-injected animals ^1, 87, 88^. This confirms a study in which young adults with depression had a positive correlation between the level of alcohol use and glutamate in the hippocampus ^89^.

### Drinking in the dark

The prototypical procedure in mice to induce binge-like drinking is giving one bottle of alcohol, offered 3 hours into the active dark photoperiod for 2–4 hours, termed drinking in the dark (DID) ( Table 1) ^90, 91^. C57BL/6J mice typically drink 2–5 g/kg in a session. Even two alcohol “binges” in adolescent rats are sufficient to abolish long-term synaptic depression in hippocampal slices and to evoke cognitive deficits via a short-lasting, repeated blockade of GluN, inducing a change in the receptor subunit composition ^92^. An earlier DID study showed that both acamprosate and MPEP decreased DID intake without affecting sugar or water drinking ^93^. Others have gone on to show that mGluR5 signaling affects PKCε in the NAc or central amgydala (CeA) to regulate DID ^94, 95^. Specifically, repeated DID for 30 days elevates CeA levels of glutamate-associated proteins of Homer2a/b, mGluR1a, GluN2B, and PLCε 24 hours after withdrawal from binge drinking ^95^. Intra-CeA and intra-NAc mGluR1 negative allosteric modulator JNJ-16259685 also reduces DID intake ^96, 97^. More recent studies have isolated downstream factors after DID such as mGluRs affecting AMPA receptor trafficking proteins like eukaryotic elongation factor 2 or decreased amygdalar CaMKIIαT286 phosphorylation ^98, 99^. Importantly, this effect was isolated to the amygdala but not NAc or dorsal striatum. This may be related to the lack of difference in frequency and amplitude of spontaneous excitatory post-synaptic current (sEPSC) in dorsolateral striatum and dorsomedial striatum medium spiny neurons between 6 weeks’ DID and water-drinking mice ^100^. Also, moving away from the classic mesolimbic pathway, others have identified a novel ventral tegmental area (VTA)–bed nucleus of the stria terminalis (BNST) CRF circuit in DID ^101^. CRF-R1 antagonists can reduce DID through intact CRF-R2 signaling, and inhibiting VTA-projecting BNST CRF neurons reduces DID ^101^. Repeated 2 g/kg alcohol injections result in enhanced GluN-mediated LTP in VTA dopamine neurons ^102^, so it is likely that this VTA-BNST glutamate pathway is altered during binge drinking in DID in a similar fashion.

Beyond DID, there are other daily limited-access procedures that lead to binge drinking in rodents. Permutations of DID exist, such as 2-hour daily access for 14 days in C57BL/6J mice, to study other facets of binge-like drinking, such as tolerance ^103^. The scheduled high alcohol consumption (SHAC) protocol involves water restriction for all but 90 minutes of water access, and every fourth day alcohol replaces water for 10–30 minutes ^104^. Systemic administration of mGluR5 antagonist MPEP decreases SHAC intake but also sucrose self-administration ^44^. Further studies have found a role for mGluR5-Homer2-PI3K signaling in the NAc in SHAC intake ^105^, which can be replicated in the DID protocol ^94^. Another limited-access protocol is multiple scheduled access (MSA), in which P rats are offered four 1-hour 2BC sessions separated by 2 hours across the dark cycle 5 days per week ^106^. Changes in gene expression in the NAc and amygdala after weeks of MSA drinking in P rats have been extensively studied ^107– 109^, so what is needed is targeting how glutamate interacts between the sites through mGluRs and iGluRs ^110^. MSA can lead to a transient increase in alcohol drinking after a weekend of deprivation ^107^, an alcohol deprivation effect (ADE), so it incorporates episodic drinking in both limited-access binge drinking and relapse-like drinking.

### Alcohol deprivation effect

Relapse is also episodic in nature, both in the clinic and modeled with animals. Relapse, a hallmark of alcohol use disorders, is the resumption of drinking following a prolonged period of abstinence. With animals, experimenters can model relapse through the expression of the ADE. In this method, alcohol-drinking animals are deprived of alcohol for a period of time (for example, days to weeks), and then following this deprivation period, an escalation in alcohol drinking is observed following re-exposure to alcohol ( Table 1). Intra-PFC glutamate and acamprosate separately reduce the ADE ^111, 112^. However, many other glutamatergic compounds—GluN/glycine receptor antagonist L-701,324, GluN2B selective antagonist ifenprodil, GluN channel blocker neramexane, GluA/GluK antagonist CNQX, and Na ^+^ channel blocker lamotrigine—attenuate the ADE similar to alcohol seeking during cue-induced reinstatement ^47, 48^. To the best of our knowledge, there are no reports for the involvement of iGluR or mGluR circuitry in the ADE, but we hypothesize that it would be similar to plastic changes in DID or operant self-administration circuitry.

It appears that episodic drinking, the amorphous transition between low-dose and high-dose intake, engages both reward-related and stress-related glutamate brain processes. A single DID protocol is mGluR5 antagonist-responsive, whereas repeated DID for a month alters changes in downstream glutamate proteins. Multiple glutamatergic compounds reduce the ADE and cue-induced reinstatement, so perhaps these protocols in combination with others would be more apt for screening medications for the clinic.

## Heavy drinking and withdrawal

Heavy drinking is defined as consuming five or more drinks on the same occasion on each of five or more days in the past month ^113^. People who exhibit heavy drinking may or may not fall into the category of mild, moderate, or severe alcohol use disorder on the basis of the accompanying psychological symptoms ^114^. As mentioned earlier, heavy drinking can be different across species. Most clinical literature focuses on alcoholics, whereas rodent studies do not have the commodity of an overarching term. For example, heavy drinking in outbred rats can be 6 g/kg per day, whereas in mice it may be 15 g/kg per day. The subsequent analysis considers heavy drinking and withdrawal for the particular species.

Tsai *et al*. ^115^ originally reported that alcohol-dependent patients have increased glutamate and glycine in the cerebrospinal fluid during withdrawal, with accompanying reduced GABA concentrations. With proton magnetic resonance spectroscopy, increased glutamate levels have been associated with more years spent drinking, loss-of-control alcohol use, and craving during detoxification in heavy drinkers or non-treatment-seeking alcoholics ^116– 118^. This glutamate dysfunction is localized to the NAc and the ACC with a positive correlation between craving and glutamate and glutamine in these regions ^118, 119^. GluN compounds like ketamine, memantine, and d-cycloserine mimic the subjective effects of alcohol in recovering alcoholics ^120– 122^. However, it is unfortunate that clinical trials with memantine or FDA-approved acamprosate did not prevent relapse compared with placebo in alcohol-dependent patients in large-scale double-blind experiments ^123– 127^. In a massive genetics study, Schumann *et al*. ^128^ reported that genetic variations in GluN2A have the greatest relevance for human alcohol dependence among 10 glutamatergic probe genes, yet increased GluN2B expression and GluN2C in the ACC and dorsolateral PFC during withdrawal can indicate likelihood of alcohol craving and risk for relapse ^129, 130^. It appears that the ACC is a distinct site for glutamate plasticity in heavy drinking.

### Intermittent access to alcohol

Cycles of binging and withdrawal occur in the transition to developing an alcohol use disorder. We can model voluntary alcohol drinking in between periods of abstinence, or alcohol deprivation, with 24-hour intermittent access to 2BC alcohol ^131– 133^. Weeks of intermittent alcohol access can lead to drinking despite adverse consequences ^134^ and signs of withdrawal such as handling-induced convulsions and decreased social interactions ^135, 136^. Giving access to alcohol for a 24-hour period may cause variability in when animals choose to drink, so researchers can also measure fluid consumption during the initial 2-, 4-, and/or 6-hour access within the 24-hour period. With this, front-loading behavior may be observed accompanied by high BACs after 2-hour access ( Table 1) ^135^. Additionally, smaller segments within 24-hour access allow drug manipulations to be assessed ^137, 138^.

Acamprosate reduces intermittent alcohol drinking in rats but not continuous-access alcohol drinking ^132^. Confirming clinical reports, outbred mice drinking on intermittent access to alcohol for 8 weeks show increased extracellular glutamate in the mPFC during withdrawal compared with 1 week of drinking and compared with water drinkers ^136^. Early reports with intermittent-access drinking in rats, drinking 7 g/kg per day, have enhanced post-synaptic GluA function in VTA neurons in the absence of any change in pre-synaptic glutamate release ^139^. Similarly, glutamatergic and GABAergic synaptic transmission are altered in the striatum of non-human primates with extended access for 3 years ^140^. Six months of continuous access and intermittent access to alcohol consumption in P rats produce selective increases in group 1 mGluR/Homer2/GluN2 expression within the NAc core and CeA ^141, 142^. Intermittent alcohol can produce short-term increases in Homer/glutamate receptor expression within both the NAc core and the CeA, which may increase the aversion of early alcohol withdrawal and consequently augment the negative reinforcing properties of alcohol. Modulators of the glutamate transporters reduce heavy drinking on a continuous-access schedule (15% and 30% ethanol) in P rats and the increased extracellular glutamate compared with water drinkers ^143^. These P rats also have enhanced expression of glutamate transporters EAAT2/GLT1 and xCT in the NAc and PFC, suggesting a role for targeting glutamate uptake in heavy drinking ^144^. Long-term intermittent alcohol recruits GABA and CRF neurons in the mPFC during withdrawal and disconnects the PFC–CeA pathway, suggesting that dysregulation of mPFC interneurons may be an early index of glutamate/GABA neuroadaptation in alcohol dependence ^145^. Impaired executive control over motivated behavior accompanies negative reinforcement during withdrawal. Seif *et al*. ^146^ show that cortical activation of NAc hyperpolarization-active GluN mediates aversion-resistant intermittent alcohol intake. Both the mPFC to NAc core and insula to NAc core mediate both quinine- and footshock-resistant alcohol drinking on an intermittent-access schedule. It appears that corticolimbic sites are integral to glutamate plasticity caused by chronic intermittent drinking.

### Withdrawal from chronic intermittent ethanol vapor and other forced alcohol methods

There are several other protocols that forcibly induce a post-dependent state in animals, such as repeated high-dose alcohol injections, alcohol liquid diet, and chronic intermittent ethanol (CIE) vapor exposure ( Table 1). Studies on brain glutamate during alcohol withdrawal have been most extensively explored using these methods, since they surpass the aversive taste of alcohol drinking solutions to induce heavy BACs. However, it is important to note that CIE is used to render rodents ethanol-dependent to subsequently increase voluntary ethanol intake, not only to maintain high BACs ^147– 149^. Microdialysis studies have shown increased glutamate in the striatum ^150^, NAc ^87, 151– 153^, and hippocampus ^1, 154^ during withdrawal in alcohol-exposed rats and mice. These results are similar to 5 g/kg alcohol gavage injections for 2–4 weeks causing increased glutamate in striatum, hippocampus, and substantia nigra 8–12 hours after the last ingestion ^155^. To counteract excitotoxicity, acamprosate and GluN antagonists have been used to decrease alcohol drinking and to alleviate symptoms of alcohol withdrawal, including increased glutamate tone and convulsive events ^112, 151, 156– 158^. It is important to note that pharmacologically increasing glutamate transmission in the NAc with TBOA, a glutamate reuptake inhibitor, can increase drinking in both non-dependent and CIE-dependent mice ^153^. Alternatively, decreasing glutamate transmission in the NAc by activating group II mGluRs reduces drinking, although the effect was stronger in dependent mice. These results comparing glutamate in non-dependent and dependent animals have similar directionality with different magnitudes, so there may be separate but overlapping actions in the NAc for treating drinking versus withdrawal symptoms with glutamatergic compounds.

In accordance with clinical studies, the PFC is a large target of glutamate plasticity in alcohol dependence. CIE results in increased GluN-mediated activity in the mPFC and increased GluN1 and GluN2B subunit expression ^159, 160^. Mice that show “compulsive-like” behaviors after CIE exhibit increased NMDA currents in the orbitofrontal cortex compared with air-exposed controls ^160^. Rescue of infralimbic PFC mGluR2 deficit restores control over alcohol-seeking behavior ^161^. It appears that mGluR2 and mGluR5 can target symptoms of withdrawal (but see ^162^). Acamprosate improved attention set-shifting of alcohol-exposed animals but did not alter the concurrent changes in synaptic transmission or membrane excitability of mPFC neurons, indicating that the changes are not the pharmacological targets of acamprosate in the recovery of mPFC functions ^163^. Abulseoud *et al*. ^164^ showed that attenuation of alcohol withdrawal by ceftriaxone induced upregulation of glutamate transporter EAAT2. Reduction of EAAT2 likely contributes to a hyperglutamatergic state in the ENT1 knockout mice ^54, 165, 166^. Some have suggested that increasing glutamate uptake through transporters has a potential therapeutic role in the treatment of alcohol dependence ^167^ (but see ^168^). Aberrations in PFC function entangle reduced executive control and poor decision making in alcoholics ^169^.

The extended amygdala—composed of the BNST, BLA, and CeA—is particularly vulnerable to glutamate plasticity caused by CIE treatment. Chronic alcohol exposure produces neuroadaptations in glutamatergic transmission in the CeA ^170, 171^, and GluN2B-containing GluNs are most sensitive to CIE ^170, 172, 173^. CIE, but not continuous vapor exposure, increases BNST GluN-mediated EPSCs, not from altered glutamate release but from an increase in GluN2-containing GluN ^174^, suggesting that repeated cycles of exposure and withdrawal are necessary for these adaptations to occur. CIE enhances long-term potentiation formation in the BNST in GluN2B knockout mice through extrasynaptic GluN ^175^. Stress-induced alterations in anxiety-like behavior were absent following bilateral infusion of GluK1 agonist ATPA into the BLA, which augmented BLA GABAergic neurotransmission, and stress increased the amplitude of sEPSC and miniature inhibitory post-synaptic current ^176^. A regulatory stress neuropeptide could be nociceptin, since nociceptin application decreases glutamate transmission and blocks alcohol-induced effects in the CeA of naïve and CIE rats, but nociceptin antagonist revealed tonic inhibitory activity of nociceptin on evoked CeA glutamatergic transmission only in alcohol-dependent rats ^177^. Changes in the extended amygdala indicate a transition from positive reinforcement to negative reinforcement as stress neuropeptides like nociceptin, CRF, and dynorphin are more engaged ^178^.

Together, chronic forced or voluntary access to alcohol affects glutamate in multiple subcortical sites like the PFC and extended amygdala, and this agrees with the clinical literature. In addition to these sites, many others have examined the hippocampus as a crux of CIE-induced glutamatergic changes. Group I mGluRs and GluN2B-containing GluNs in CA1 and cortex impair LTD, reduce spine density, and disrupt learning ^179, 180^ (but see 138). This may be related to the enhanced stress systems recruited during repeated exposure to and withdrawal from alcohol. In line with this hypothesis, corticohippocampal GluN2B is engaged during repeated swim stress ^181^. This circuitry is also recruited in other addictive disorders. Glutamate homeostasis is a mediator of long-term drug-seeking behavior, especially through disruptions of the cysteine/glutamate exchanger and EAAT2/GLT1 ^182^. Alterations in glutamate transmission after chronic alcohol exposure and withdrawal are evident, but some effects are also likely to be unique to withdrawal alone. Future research can tease apart these dynamic distinctions or suggest that they are interconnected.

## Discussion

**Figure 1.**
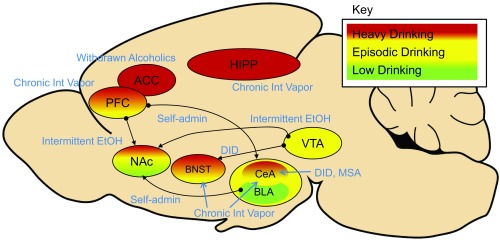
A sagittal representation of subcortical structures and their circuitry related to different stages during the transition from low-level drinking to heavy alcohol use. Regions of interest in red indicate involvement in heavy drinking, yellow in episodic drinking, and green in lower-level drinking. Known connections start with the black circle and finish with the black arrowhead. Animal drinking protocols are depicted in blue italics. ACC, anterior cingulate cortex; BLA, basolateral amygdala; BNST, bed nucleus of the stria terminalis; CeA, central amygdala; HIPP, hippocampus; NAc, nucleus accumbens; PFC, prefrontal cortex; VTA, ventral tegmental area.

Across all phases of alcohol drinking, glutamate is a critical regulator of subcortical plasticity in the brain. We have mapped some relevant regions of interest according to their involvement in low, moderate, or heavy drinking ( Figure 1), but more work can be done to study how these sites work on a circuit level. Downstream signaling factors like CaMKII are important in the PFC and amygdala in operant self-administration. Binge drinking in the DID protocol also affects mGluR5 in the CeA and CRF in the BNST in connection with mesolimbic targets. Glutamate in the ACC and PFC is heavily disrupted in alcoholics, which is supported by preclinical research using intermittent access to alcohol or CIE. Electrophysiological studies also reveal a role for GluN2 in the extended amygdala in alcohol withdrawal, related to negative affect. Furthermore, glutamate transmission in circuits stemming from the NAc represents an overlap in circuitry from light to episodic to heavy drinking in a limited-access model. The roles of glutamate transporters and the interaction with glia are better understood at both ends of the drinking spectrum (2BC and CIE), but more can be learned through intermediate protocols that reveal the transition to heavy drinking. Overall, there appears to be a shift from mGluR-dependent behaviors to GluN-related processes transitioning from lower-level alcohol drinking to heavy alcohol drinking. The efficacy of acamprosate in animal models of drinking is high, in sharp contrast to its ineffective treatment in the clinic. Therefore, research needs to focus on other promising glutamatergic compounds to reduce heavy drinking or mediate withdrawal symptoms or both.

## References

[1] Chefer V Meis J Wang G: Repeated exposure to moderate doses of ethanol augments hippocampal glutamate neurotransmission by increasing release. *Addict Biol.* 2011;16(2):229–37. 10.1111/j.1369-1600.2010.00272.x 21182572 PMC3684957

[2] Rossetti ZL Carboni S Fadda F: Glutamate-induced increase of extracellular glutamate through *N*-methyl-D-aspartate receptors in ethanol withdrawal. *Neuroscience.* 1999;93(3):1135–40. 10.1016/S0306-4522(99)00250-X 10473277

[3] Poldrugo F Snead OC 3rd: Electroencephalographic and behavioral correlates in rats during repeated ethanol withdrawal syndromes. *Psychopharmacology (Berl).* 1984;83(2):140–6. 10.1007/BF00429722 6431463

[4] Becker HC Hale RL: Repeated episodes of ethanol withdrawal potentiate the severity of subsequent withdrawal seizures: an animal model of alcohol withdrawal "kindling". *Alcohol Clin Exp Res.* 1993;17(1):94–8. 10.1111/j.1530-0277.1993.tb00731.x 8452212

[5] Ballenger JC Post RM: Kindling as a model for alcohol withdrawal syndromes. *Br J Psychiatry.* 1978;133(1):1–14. 10.1192/bjp.133.1.1 352467

[6] Holmes A Spanagel R Krystal JH: Glutamatergic targets for new alcohol medications. *Psychopharmacology (Berl).* 2013;229(3):539–54. 10.1007/s00213-013-3226-2 23995381 PMC3811052

[7] Selim M Bradberry CW: Effect of ethanol on extracellular 5-HT and glutamate in the nucleus accumbens and prefrontal cortex: comparison between the Lewis and Fischer 344 rat strains. *Brain Res.* 1996;716(1–2):157–64. 10.1016/0006-8993(95)01385-7 8738232

[8] Allgaier C: Ethanol sensitivity of NMDA receptors. *Neurochem Int.* 2002;41(6):377–82. 10.1016/S0197-0186(02)00046-3 12213224

[9] Lovinger DM White G Weight FF: Ethanol inhibits NMDA-activated ion current in hippocampal neurons. *Science.* 1989;243(4899):1721–4. 10.1126/science.2467382 2467382

[10] Mirshahi T Woodward JJ: Ethanol sensitivity of heteromeric NMDA receptors: effects of subunit assembly, glycine and NMDAR1 Mg ^2+^-insensitive mutants. *Neuropharmacology.* 1995;34(3):347–55. 10.1016/0028-3908(94)00155-L 7630488

[11] Kähkönen S Wilenius J Nikulin VV: Alcohol reduces prefrontal cortical excitability in humans: a combined TMS and EEG study. *Neuropsychopharmacology.* 2003;28(4):747–54. 10.1038/sj.npp.1300099 12655321

[12] Tu Y Kroener S Abernathy K: Ethanol inhibits persistent activity in prefrontal cortical neurons. *J Neurosci.* 2007;27(17):4765–75. 10.1523/JNEUROSCI.5378-06.2007 17460089 PMC3625968

[13] McMillen BA Joyner PW Parmar CA: Effects of NMDA glutamate receptor antagonist drugs on the volitional consumption of ethanol by a genetic drinking rat. *Brain Res Bull.* 2004;64(3):279–84. 10.1016/j.brainresbull.2004.08.001 15464866

[14] Chen YW Barson JR Chen A: Glutamatergic input to the lateral hypothalamus stimulates ethanol intake: role of orexin and melanin-concentrating hormone. *Alcohol Clin Exp Res.* 2013;37(1):123–31. 10.1111/j.1530-0277.2012.01854.x 22823322 PMC4007270

[15] Lockridge A Romero G Harrington J: Timing-dependent reduction in ethanol sedation and drinking preference by NMDA receptor co-agonist d-serine. *Alcohol.* 2012;46(4):389–400. 10.1016/j.alcohol.2011.11.004 22445805

[16] Debrouse L Hurd B Kiselycznyk C: Probing the modulation of acute ethanol intoxication by pharmacological manipulation of the NMDAR glycine co-agonist site. *Alcohol Clin Exp Res.* 2013;37(2):223–33. 10.1111/j.1530-0277.2012.01922.x 22934986 PMC3515721

[17] Boyce-Rustay JM Holmes A: Ethanol-related behaviors in mice lacking the NMDA receptor NR2A subunit. *Psychopharmacology (Berl).* 2006;187(4):455–66. 10.1007/s00213-006-0448-6 16835771

[18] Cowen MS Schroff KC Gass P: Neurobehavioral effects of alcohol in AMPA receptor subunit (GluR1) deficient mice. *Neuropharmacology.* 2003;45(3):325–33. 10.1016/S0028-3908(03)00174-6 12871650

[19] Kiefer F Jahn H Koester A: Involvement of NMDA receptors in alcohol-mediated behavior: mice with reduced affinity of the NMDA R1 glycine binding site display an attenuated sensitivity to ethanol. *Biol Psychiatry.* 2003;53(4):345–51. 10.1016/S0006-3223(02)01486-5 12586454

[20] Blednov YA Harris RA: Metabotropic glutamate receptor 5 (mGluR5) regulation of ethanol sedation, dependence and consumption: relationship to acamprosate actions. *Int J Neuropsychopharmacol.* 2008;11(6):775–93. 10.1017/S1461145708008584 18377703 PMC2574715

[21] Bird MK Kirchhoff J Djouma E: Metabotropic glutamate 5 receptors regulate sensitivity to ethanol in mice. *Int J Neuropsychopharmacol.* 2008;11(6):765–74. 10.1017/S1461145708008572 18400131

[22] McMillen BA Crawford MS Kulers CM: Effects of a metabotropic, mglu5, glutamate receptor antagonist on ethanol consumption by genetic drinking rats. *Alcohol Alcohol.* 2005;40(6):494–7. 10.1093/alcalc/agh200 16186143

[23] Cowen MS Djouma E Lawrence AJ: The metabotropic glutamate 5 receptor antagonist 3-[(2-methyl-1,3-thiazol-4-yl)ethynyl]-pyridine reduces ethanol self-administration in multiple strains of alcohol-preferring rats and regulates olfactory glutamatergic systems. *J Pharmacol Exp Ther.* 2005;315(2):590–600. 10.1124/jpet.105.090449 16014750

[24] Bahi A Fizia K Dietz M: Pharmacological modulation of mGluR7 with AMN082 and MMPIP exerts specific influences on alcohol consumption and preference in rats. *Addict Biol.* 2012;17(2):235–47. 10.1111/j.1369-1600.2010.00310.x 21392179

[25] Bahi A: Viral-mediated knockdown of mGluR7 in the nucleus accumbens mediates excessive alcohol drinking and increased ethanol-elicited conditioned place preference in rats. *Neuropsychopharmacology.* 2013;38(11):2109–19. 10.1038/npp.2012.122 22781839 PMC3773660

[26] Szumlinski KK Lominac KD Oleson EB: Homer2 is necessary for EtOH-induced neuroplasticity. *J Neurosci.* 2005;25(30):7054–61. 10.1523/JNEUROSCI.1529-05.2005 16049182 PMC6724845

[27] Boismare F Daoust M Moore N: A homotaurine derivative reduces the voluntary intake of ethanol by rats: are cerebral GABA receptors involved? *Pharmacol Biochem Behav.* 1984;21(5):787–9. 10.1016/s0091-3057(84)80020-9 6096898

[28] Yoneyama N Crabbe JC Ford MM: Voluntary ethanol consumption in 22 inbred mouse strains. *Alcohol.* 2008;42(3):149–60. 10.1016/j.alcohol.2007.12.006 18358676 PMC2396347

[29] McCool BA Chappell AM: Persistent enhancement of ethanol drinking following a monosodium glutamate-substitution procedure in C57BL6/J and DBA/2J mice. *Alcohol.* 2014;48(1):55–61. 10.1016/j.alcohol.2013.10.008 24355071 PMC3946935

[30] Fidler TL Dion AM Powers MS: Intragastric self-infusion of ethanol in high- and low-drinking mouse genotypes after passive ethanol exposure. *Genes Brain Behav.* 2011;10(3):264–75. 10.1111/j.1601-183X.2010.00664.x 21091635 PMC3070055

[31] Wang Z Chow SY: Effects of glutamate, *N*-methyl- D-aspartate, high potassium, and hypoxia on unit discharges in CA1 area of hippocampal slices of DBA and C57 mice. *Epilepsia.* 1995;36(2):196–206. 10.1111/j.1528-1157.1995.tb00980.x 7821278

[32] Wanat MJ Sparta DR Hopf FW: Strain specific synaptic modifications on ventral tegmental area dopamine neurons after ethanol exposure. *Biol Psychiatry.* 2009;65(8):646–53. 10.1016/j.biopsych.2008.10.042 19118821 PMC3040034

[33] Kerns RT Ravindranathan A Hassan S: Ethanol-responsive brain region expression networks: implications for behavioral responses to acute ethanol in DBA/2J versus C57BL/6J mice. *J Neurosci.* 2005;25(9):2255–66. 10.1523/JNEUROSCI.4372-04.2005 15745951 PMC6726093

[34] Zhou Z Karlsson C Liang T: Loss of metabotropic glutamate receptor 2 escalates alcohol consumption. *Proc Natl Acad Sci U S A.* 2013;110(42):16963–8. 10.1073/pnas.1309839110 24082084 PMC3800985

[35] Sabino V Narayan AR Zeric T: mTOR activation is required for the anti-alcohol effect of ketamine, but not memantine, in alcohol-preferring rats. *Behav Brain Res.* 2013;247:9–16. 10.1016/j.bbr.2013.02.030 23466691 PMC3646912

[36] Hodge CW Miles MF Sharko AC: The mGluR5 antagonist MPEP selectively inhibits the onset and maintenance of ethanol self-administration in C57BL/6J mice. *Psychopharmacology (Berl).* 2006;183(4):429–38. 10.1007/s00213-005-0217-y 16292590 PMC2854492

[37] Schroeder JP Overstreet DH Hodge CW: The mGluR5 antagonist MPEP decreases operant ethanol self-administration during maintenance and after repeated alcohol deprivations in alcohol-preferring (P) rats. *Psychopharmacology (Berl).* 2005;179(1):262–70. 10.1007/s00213-005-2175-9 15717208 PMC11583314

[38] Cowen MS Krstew E Lawrence AJ: Assessing appetitive and consummatory phases of ethanol self-administration in C57BL/6J mice under operant conditions: regulation by mGlu5 receptor antagonism. *Psychopharmacology (Berl).* 2007;190(1):21–9. 10.1007/s00213-006-0583-0 17096086

[39] Salling MC Faccidomo S Hodge CW: Nonselective suppression of operant ethanol and sucrose self-administration by the mGluR7 positive allosteric modulator AMN082. *Pharmacol Biochem Behav.* 2008;91(1):14–20. 10.1016/j.pbb.2008.06.006 18593591 PMC2562464

[40] Adams CL Cowen MS Short JL: Combined antagonism of glutamate mGlu5 and adenosine A2A receptors interact to regulate alcohol-seeking in rats. *Int J Neuropsychopharmacol.* 2008;11(2):229–41. 10.1017/S1461145707007845 17517168

[41] Besheer J Faccidomo S Grondin JJ: Regulation of motivation to self-administer ethanol by mGluR5 in alcohol-preferring (P) rats. *Alcohol Clin Exp Res.* 2008;32(2):209–21. 10.1111/j.1530-0277.2007.00570.x 18162077 PMC2532085

[42] Besheer J Grondin JJ Cannady R: Metabotropic glutamate receptor 5 activity in the nucleus accumbens is required for the maintenance of ethanol self-administration in a rat genetic model of high alcohol intake. *Biol Psychiatry.* 2010;67(9):812–22. 10.1016/j.biopsych.2009.09.016 19897175 PMC2854174

[43] Sharko AC Hodge CW: Differential modulation of ethanol-induced sedation and hypnosis by metabotropic glutamate receptor antagonists in C57BL/6J mice. *Alcohol Clin Exp Res.* 2008;32(1):67–76. 10.1111/j.1530-0277.2007.00554.x 18070246 PMC2533745

[44] Tanchuck MA Yoneyama N Ford MM: Assessment of GABA-B, metabotropic glutamate, and opioid receptor involvement in an animal model of binge drinking. *Alcohol.* 2011;45(1):33–44. 10.1016/j.alcohol.2010.07.009 20843635 PMC3003942

[45] Parkitna JR Sikora M Gołda S: Novelty-seeking behaviors and the escalation of alcohol drinking after abstinence in mice are controlled by metabotropic glutamate receptor 5 on neurons expressing dopamine d1 receptors. *Biol Psychiatry.* 2013;73(3):263–70. 10.1016/j.biopsych.2012.07.019 22902169

[46] Shaham Y Shalev U Lu L: The reinstatement model of drug relapse: history, methodology and major findings. *Psychopharmacology (Berl).* 2003;168(1–2):3–20. 10.1007/s00213-002-1224-x 12402102

[47] Bäckström P Hyytiä P: Ionotropic glutamate receptor antagonists modulate cue-induced reinstatement of ethanol-seeking behavior. *Alcohol Clin Exp Res.* 2004;28(4):558–65. 10.1097/01.ALC.0000122101.13164.21 15100606

[48] Vengeliene V Bachteler D Danysz W: The role of the NMDA receptor in alcohol relapse: a pharmacological mapping study using the alcohol deprivation effect. *Neuropharmacology.* 2005;48(6):822–9. 10.1016/j.neuropharm.2005.01.002 15829254

[49] Bäckström P Bachteler D Koch S: mGluR5 antagonist MPEP reduces ethanol-seeking and relapse behavior. *Neuropsychopharmacology.* 2004;29(5):921–8. 10.1038/sj.npp.1300381 14735132

[50] Bäckström P Hyytiä P: Suppression of alcohol self-administration and cue-induced reinstatement of alcohol seeking by the mGlu2/3 receptor agonist LY379268 and the mGlu8 receptor agonist (S)-3,4-DCPG. *Eur J Pharmacol.* 2005;528(1–3):110–8. 10.1016/j.ejphar.2005.10.051 16324694

[51] Rodd ZA McKinzie DL Bell RL: The metabotropic glutamate 2/3 receptor agonist LY404039 reduces alcohol-seeking but not alcohol self-administration in alcohol-preferring (P) rats. *Behav Brain Res.* 2006;171(2):207–15. 10.1016/j.bbr.2006.03.032 16678921

[52] Kufahl PR Martin-Fardon R Weiss F: Enhanced sensitivity to attenuation of conditioned reinstatement by the mGluR _2/3_ agonist LY379268 and increased functional activity of mGluR _2/3_ in rats with a history of ethanol dependence. *Neuropsychopharmacology.* 2011;36(13):2762–73. 10.1038/npp.2011.174 21881571 PMC3230501

[53] Gass JT Sinclair CM Cleva RM: Alcohol-seeking behavior is associated with increased glutamate transmission in basolateral amygdala and nucleus accumbens as measured by glutamate-oxidase-coated biosensors. *Addict Biol.* 2011;16(2):215–28. 10.1111/j.1369-1600.2010.00262.x 21054692 PMC3058760

[54] Chen J Nam HW Lee MR: Altered glutamatergic neurotransmission in the striatum regulates ethanol sensitivity and intake in mice lacking ENT1. *Behav Brain Res.* 2010;208(2):636–42. 10.1016/j.bbr.2010.01.011 20085785 PMC2831139

[55] Weiland A Garcia S Knackstedt LA: Ceftriaxone and cefazolin attenuate the cue-primed reinstatement of alcohol-seeking. *Front Pharmacol.* 2015;6:44. 10.3389/fphar.2015.00044 25805996 PMC4354333

[56] Olive MF McGeehan AJ Kinder JR: The mGluR5 antagonist 6-methyl-2-(phenylethynyl)pyridine decreases ethanol consumption via a protein kinase C epsilon-dependent mechanism. *Mol Pharmacol.* 2005;67(2):349–55. 10.1124/mol.104.003319 15548766

[57] Faccidomo S Besheer J Stanford PC: Increased operant responding for ethanol in male C57BL/6J mice: specific regulation by the ERK _1/2_, but not JNK, MAP kinase pathway. *Psychopharmacology (Berl).* 2009;204(1):135–47. 10.1007/s00213-008-1444-9 19125235 PMC2845162

[58] Faccidomo S Salling MC Galunas C: Operant ethanol self-administration increases extracellular-signal regulated protein kinase (ERK) phosphorylation in reward-related brain regions: selective regulation of positive reinforcement in the prefrontal cortex of C57BL/6J mice. *Psychopharmacology (Berl).* 2015;232(18):3417–30. 10.1007/s00213-015-3993-z 26123321 PMC4537834

[59] Schroeder JP Spanos M Stevenson JR: Cue-induced reinstatement of alcohol-seeking behavior is associated with increased ERK _1/2_ phosphorylation in specific limbic brain regions: blockade by the mGluR5 antagonist MPEP. *Neuropharmacology.* 2008;55(4):546–54. 10.1016/j.neuropharm.2008.06.057 18619984 PMC2613007

[60] Cannady R Fisher KR Durant B: Enhanced AMPA receptor activity increases operant alcohol self-administration and cue-induced reinstatement. *Addict Biol.* 2013;18(1):54–65. 10.1111/adb.12000 23126443 PMC3535558

[61] Cannady R Fisher KR Graham C: Potentiation of amygdala AMPA receptor activity selectively promotes escalated alcohol self-administration in a CaMKII-dependent manner. *Addict Biol.* 2016. 10.1111/adb.12357 26742808 PMC4935658

[62] Salling MC Faccidomo SP Li C: Moderate Alcohol Drinking and the Amygdala Proteome: Identification and Validation of Calcium/Calmodulin Dependent Kinase II and AMPA Receptor Activity as Novel Molecular Mechanisms of the Positive Reinforcing Effects of Alcohol. *Biol Psychiatry.* 2016;79(6):430–42. 10.1016/j.biopsych.2014.10.020 25579851 PMC4417085

[63] Faccidomo S Reid GT Agoglia AE: CaMKII inhibition in the prefrontal cortex specifically increases the positive reinforcing effects of sweetened alcohol in C57BL/6J mice. *Behav Brain Res.* 2016;298(Pt B):286–90. 10.1016/j.bbr.2015.11.018 26608538 PMC4688209

[64] Grant KA: Strategies for understanding the pharmacological effects of ethanol with drug discrimination procedures. *Pharmacol Biochem Behav.* 1999;64(2):261–7. 10.1016/S0091-3057(99)00075-1 10515301

[65] Kostowski W Bieńkowski P: Discriminative stimulus effects of ethanol: neuropharmacological characterization. *Alcohol.* 1999;17(1):63–80. 10.1016/S0741-8329(98)00035-4 9895039

[66] Hodge CW Cox AA: The discriminative stimulus effects of ethanol are mediated by NMDA and GABA(A) receptors in specific limbic brain regions. *Psychopharmacology (Berl).* 1998;139(1–2):95–107. 10.1007/s002130050694 9768547

[67] Hundt W Danysz W Hölter SM: Ethanol and N-methyl-D-aspartate receptor complex interactions: a detailed drug discrimination study in the rat. *Psychopharmacology (Berl).* 1998;135(1):44–51. 10.1007/s002130050484 9489933

[68] Vivian JA Waters CA Szeliga KT: Characterization of the discriminative stimulus effects of N-methyl- D-aspartate ligands under different ethanol training conditions in the cynomolgus monkey ( Macaca fascicularis). *Psychopharmacology (Berl).* 2002;162(3):273–81. 10.1007/s00213-002-1086-2 12122485

[69] Spanagel R Zieglgänsberger W Hundt W: Acamprosate and alcohol: III. Effects on alcohol discrimination in the rat. *Eur J Pharmacol.* 1996;305(1–3):51–6. 10.1016/0014-2999(96)00176-8 8813531

[70] Besheer J Cox AA Hodge CW: Coregulation of ethanol discrimination by the nucleus accumbens and amygdala. *Alcohol Clin Exp Res.* 2003;27(3):450–6. 10.1097/01.ALC.0000057036.64169.C1 12658110

[71] Besheer J Stevenson RA Hodge CW: mGlu _5_ receptors are involved in the discriminative stimulus effects of self-administered ethanol in rats. *Eur J Pharmacol.* 2006;551(1–3):71–5. 10.1016/j.ejphar.2006.08.071 17026991 PMC1676072

[72] Besheer J Grondin JJ Salling MC: Interoceptive effects of alcohol require mGlu5 receptor activity in the nucleus accumbens. *J Neurosci.* 2009;29(30):9582–91. 10.1523/JNEUROSCI.2366-09.2009 19641121 PMC2845172

[73] Cannady R Grondin JJ Fisher KR: Activation of group II metabotropic glutamate receptors inhibits the discriminative stimulus effects of alcohol via selective activity within the amygdala. *Neuropsychopharmacology.* 2011;36(11):2328–38. 10.1038/npp.2011.121 21734651 PMC3176569

[74] Besheer J Fisher KR Cannady R: Intra-amygdala inhibition of ERK _1/2_ potentiates the discriminative stimulus effects of alcohol. *Behav Brain Res.* 2012;228(2):398–405. 10.1016/j.bbr.2011.12.023 22209853 PMC3268949

[75] Besheer J Fisher KR Jaramillo AA: Stress hormone exposure reduces mGluR5 expression in the nucleus accumbens: functional implications for interoceptive sensitivity to alcohol. *Neuropsychopharmacology.* 2014;39(10):2376–86. 10.1038/npp.2014.85 24713611 PMC4138747

[76] Jaramillo AA Randall PA Frisbee S: Activation of mGluR2/3 following stress hormone exposure restores sensitivity to alcohol in rats. *Alcohol.* 2015;49(6):525–32. 10.1016/j.alcohol.2015.03.008 26142564 PMC4554830

[77] Jaramillo AA Randall PA Frisbee S: Modulation of sensitivity to alcohol by cortical and thalamic brain regions. *Eur J Neurosci.* 2016;44(8):2569–80. 10.1111/ejn.13374 27543844 PMC5377065

[78] Brigman JL Daut RA Wright T: GluN2B in corticostriatal circuits governs choice learning and choice shifting. *Nat Neurosci.* 2013;16(8):1101–10. 10.1038/nn.3457 23831965 PMC3725191

[79] Fu R Zuo W Gregor D: Pharmacological Manipulation of the Rostromedial Tegmental Nucleus Changes Voluntary and Operant Ethanol Self-Administration in Rats. *Alcohol Clin Exp Res.* 2016;40(3):572–82. 10.1111/acer.12974 26876382 PMC4775316

[80] Miczek KA Barros HM Sakoda L: Alcohol and heightened aggression in individual mice. *Alcohol Clin Exp Res.* 1998;22(8):1698–705. 10.1111/j.1530-0277.1998.tb03968.x 9835283

[81] Newman EL Chu A Bahamon B: NMDA receptor antagonism: escalation of aggressive behavior in alcohol-drinking mice. *Psychopharmacology (Berl).* 2012;224(1):167–77. 10.1007/s00213-012-2734-9 22588250 PMC3694321

[82] Quadros IM Hwa LS Shimamoto A: Prevention of alcohol-heightened aggression by CRF-R1 antagonists in mice: critical role for DRN-PFC serotonin pathway. *Neuropsychopharmacology.* 2014;39(12):2874–83. 10.1038/npp.2014.139 24917195 PMC4200498

[83] Naimi TS Brewer RD Mokdad A: Binge drinking among US adults. *JAMA.* 2003;289(1):70–5. 10.1001/jama.289.1.70 12503979

[84] Bates ME Labouvie EW: Adolescent risk factors and the prediction of persistent alcohol and drug use into adulthood. *Alcohol Clin Exp Res.* 1997;21(5):944–50. 10.1111/j.1530-0277.1997.tb03863.x 9267549

[85] Lee E Jang DP Kim JJ: Alteration of brain metabolites in young alcoholics without structural changes. *Neuroreport.* 2007;18(14):1511–4. 10.1097/WNR.0b013e3282ef7625 17712285

[86] Silveri MM Cohen-Gilbert J Crowley DJ: Altered anterior cingulate neurochemistry in emerging adult binge drinkers with a history of alcohol-induced blackouts. *Alcohol Clin Exp Res.* 2014;38(4):969–79. 10.1111/acer.12346 24512596 PMC4465537

[87] Kapasova Z Szumlinski KK: Strain differences in alcohol-induced neurochemical plasticity: a role for accumbens glutamate in alcohol intake. *Alcohol Clin Exp Res.* 2008;32(4):617–31. 10.1111/j.1530-0277.2008.00620.x 18341649

[88] Ward RJ Colivicchi MA Allen R: Neuro-inflammation induced in the hippocampus of 'binge drinking' rats may be mediated by elevated extracellular glutamate content. *J Neurochem.* 2009;111(5):1119–28. 10.1111/j.1471-4159.2009.06389.x 19765190

[89] Hermens DF Chitty KM Lee RS: Hippocampal glutamate is increased and associated with risky drinking in young adults with major depression. *J Affect Disord.* 2015;186:95–8. 10.1016/j.jad.2015.07.009 26233319

[90] Rhodes JS Best K Belknap JK: Evaluation of a simple model of ethanol drinking to intoxication in C57BL/6J mice. *Physiol Behav.* 2005;84(1):53–63. 10.1016/j.physbeh.2004.10.007 15642607

[91] Thiele TE Crabbe JC Boehm SL 2nd: "Drinking in the Dark" (DID): a simple mouse model of binge-like alcohol intake. *Curr Protoc Neurosci.* 2014;68:9.49.1–12. 10.1002/0471142301.ns0949s68 24984686 PMC4142649

[92] Silvestre de Ferron B Bennouar KE Kervern M: Two Binges of Ethanol a Day Keep the Memory Away in Adolescent Rats: Key Role for GLUN2B Subunit. *Int J Neuropsychopharmacol.* 2015;19(1): pii: pyv087. 10.1093/ijnp/pyv087 26254123 PMC4772273

[93] Gupta T Syed YM Revis AA: Acute effects of acamprosate and MPEP on ethanol Drinking-in-the-Dark in male C57BL/6J mice. *Alcohol Clin Exp Res.* 2008;32(11):1992–8. 10.1111/j.1530-0277.2008.00787.x 18782337

[94] Cozzoli DK Courson J Caruana AL: Nucleus accumbens mGluR5-associated signaling regulates binge alcohol drinking under drinking-in-the-dark procedures. *Alcohol Clin Exp Res.* 2012;36(9):1623–33. 10.1111/j.1530-0277.2012.01776.x 22432643 PMC3382009

[95] Cozzoli DK Courson J Rostock C: Protein Kinase C Epsilon Activity in the Nucleus Accumbens and Central Nucleus of the Amygdala Mediates Binge Alcohol Consumption. *Biol Psychiatry.* 2016;79(6):443–51. 10.1016/j.biopsych.2015.01.019 25861702 PMC4561036

[96] Cozzoli DK Courson J Wroten MG: Binge alcohol drinking by mice requires intact group1 metabotropic glutamate receptor signaling within the central nucleus of the amygdala. *Neuropsychopharmacology.* 2014;39(2):435–44. 10.1038/npp.2013.214 23966068 PMC3870786

[97] Lum EN Campbell RR Rostock C: mGluR1 within the nucleus accumbens regulates alcohol intake in mice under limited-access conditions. *Neuropharmacology.* 2014;79:679–87. 10.1016/j.neuropharm.2014.01.024 24467847 PMC3957427

[98] Meyers JL Salling MC Almli LM: Frequency of alcohol consumption in humans; the role of metabotropic glutamate receptors and downstream signaling pathways. *Transl Psychiatry.* 2015;5(6):e586. 10.1038/tp.2015.70 26101849 PMC4490281

[99] Agoglia AE Holstein SE Reid G: CaMKIIalpha-GluA1 Activity Underlies Vulnerability to Adolescent Binge Alcohol Drinking. *Alcohol Clin Exp Res.* 2015;39(9):1680–90. 10.1111/acer.12819 26247621 PMC4558330

[100] Wilcox MV Cuzon Carlson VC Sherazee N: Repeated binge-like ethanol drinking alters ethanol drinking patterns and depresses striatal GABAergic transmission. *Neuropsychopharmacology.* 2014;39(3):579–94. 10.1038/npp.2013.230 23995582 PMC3895236

[101] Rinker JA Marshall SA Mazzone CM: Extended Amygdala to Ventral Tegmental Area Corticotropin-Releasing Factor Circuit Controls Binge Ethanol Intake. *Biol Psychiatry.* 2016; pii: S0006-3223(16)30006-3. 10.1016/j.biopsych.2016.02.029 27113502 PMC5010800

[102] Bernier BE Whitaker LR Morikawa H: Previous ethanol experience enhances synaptic plasticity of NMDA receptors in the ventral tegmental area. *J Neurosci.* 2011;31(14):5205–12. 10.1523/JNEUROSCI.5282-10.2011 21471355 PMC3086894

[103] Linsenbardt DN Moore EM Griffin KD: Tolerance to ethanol's ataxic effects and alterations in ethanol-induced locomotion following repeated binge-like ethanol intake using the DID model. *Alcohol Clin Exp Res.* 2011;35(7):1246–55. 10.1111/j.1530-0277.2011.01459.x 21410484 PMC3117122

[104] Finn DA Belknap JK Cronise K: A procedure to produce high alcohol intake in mice. *Psychopharmacology (Berl).* 2005;178(4):471–80. 10.1007/s00213-004-2039-8 15765261

[105] Cozzoli DK Goulding SP Zhang PW: Binge drinking upregulates accumbens mGluR5-Homer2-PI3K signaling: functional implications for alcoholism. *J Neurosci.* 2009;29(27):8655–68. 10.1523/JNEUROSCI.5900-08.2009 19587272 PMC2761716

[106] Murphy JM Gatto GJ Waller MB: Effects of scheduled access on ethanol intake by the alcohol-preferring (P) line of rats. *Alcohol.* 1986;3(5):331–6. 10.1016/0741-8329(86)90010-8 3778650

[107] Bell RL Kimpel MW Rodd ZA: Protein expression changes in the nucleus accumbens and amygdala of inbred alcohol-preferring rats given either continuous or scheduled access to ethanol. *Alcohol.* 2006;40(1):3–17. 10.1016/j.alcohol.2006.10.001 17157716

[108] Bell RL Kimpel MW McClintick JN: Gene expression changes in the nucleus accumbens of alcohol-preferring rats following chronic ethanol consumption. *Pharmacol Biochem Behav.* 2009;94(1):131–47. 10.1016/j.pbb.2009.07.019 19666046 PMC2771758

[109] McBride WJ Kimpel MW Schultz JA: Changes in gene expression in regions of the extended amygdala of alcohol-preferring rats after binge-like alcohol drinking. *Alcohol.* 2010;44(2):171–83. 10.1016/j.alcohol.2009.12.001 20116196 PMC2831121

[110] Bell RL Hauser SR McClintick J: Ethanol-Associated Changes in Glutamate Reward Neurocircuitry: A Minireview of Clinical and Preclinical Genetic Findings. *Prog Mol Biol Transl Sci.* 2016;137:41–85. 10.1016/bs.pmbts.2015.10.018 26809998 PMC4749142

[111] Salimov RM Salimova NB: The alcohol-deprivation effect in hybrid mice. *Drug Alcohol Depend.* 1993;32(2):187–91. 10.1016/0376-8716(93)80012-4 8508729

[112] Spanagel R Hölter SM Allingham K: Acamprosate and alcohol: I. Effects on alcohol intake following alcohol deprivation in the rat. *Eur J Pharmacol.* 1996;305(1–3):39–44. 10.1016/0014-2999(96)00174-4 8813529

[113] (SAMHSA), S. A. and M. H. S. A: 2014 National Survey on Drug Use and Health (NSDUH). 2014 Reference Source

[114] Association A.P: Diagnostic and Statistical Manual of Mental Disorders. 2013 Reference Source

[115] Tsai GE Ragan P Chang R: Increased glutamatergic neurotransmission and oxidative stress after alcohol withdrawal. *Am J Psychiatry.* 1998;155(6):726–32. 9619143 10.1176/ajp.155.6.726

[116] Yeo RA Thoma RJ Gasparovic C: Neurometabolite concentration and clinical features of chronic alcohol use: a proton magnetic resonance spectroscopy study. *Psychiatry Res.* 2013;211(2):141–7. 10.1016/j.pscychresns.2012.05.005 23154093 PMC3570754

[117] Ende G Hermann D Demirakca T: Loss of control of alcohol use and severity of alcohol dependence in non-treatment-seeking heavy drinkers are related to lower glutamate in frontal white matter. *Alcohol Clin Exp Res.* 2013;37(10):1643–9. 10.1111/acer.12149 23800328

[118] Bauer J Pedersen A Scherbaum N: Craving in alcohol-dependent patients after detoxification is related to glutamatergic dysfunction in the nucleus accumbens and the anterior cingulate cortex. *Neuropsychopharmacology.* 2013;38(8):1401–8. 10.1038/npp.2013.45 23403696 PMC3682141

[119] Mon A Durazzo TC Meyerhoff DJ: Glutamate, GABA, and other cortical metabolite concentrations during early abstinence from alcohol and their associations with neurocognitive changes. *Drug Alcohol Depend.* 2012;125(1–2):27–36. 10.1016/j.drugalcdep.2012.03.012 22503310 PMC3419314

[120] Krystal JH Petrakis IL Webb E: Dose-related ethanol-like effects of the NMDA antagonist, ketamine, in recently detoxified alcoholics. *Arch Gen Psychiatry.* 1998;55(4):354–60. 10.1001/archpsyc.55.4.354 9554431

[121] Krystal JH Petrakis IL Limoncelli D: Altered NMDA glutamate receptor antagonist response in recovering ethanol-dependent patients. *Neuropsychopharmacology.* 2003;28(11):2020–8. 10.1038/sj.npp.1300252 12888778

[122] Krystal JH Petrakis IL Limoncelli D: Characterization of the interactive effects of glycine and D-cycloserine in men: further evidence for enhanced NMDA receptor function associated with human alcohol dependence. *Neuropsychopharmacology.* 2011;36(3):701–10. 10.1038/npp.2010.203 21124304 PMC3055693

[123] Evans SM Levin FR Brooks DJ: A pilot double-blind treatment trial of memantine for alcohol dependence. *Alcohol Clin Exp Res.* 2007;31(5):775–82. 10.1111/j.1530-0277.2007.00360.x 17378918

[124] Anton RF O'Malley SS Ciraulo DA: Combined pharmacotherapies and behavioral interventions for alcohol dependence: the COMBINE study: a randomized controlled trial. *JAMA.* 2006;295(17):2003–17. 10.1001/jama.295.17.2003 16670409

[125] Mann K Lemenager T Hoffmann S: Results of a double-blind, placebo-controlled pharmacotherapy trial in alcoholism conducted in Germany and comparison with the US COMBINE study. *Addict Biol.* 2013;18(6):937–46. 10.1111/adb.12012 23231446

[126] Bisaga A Evans SM: Acute effects of memantine in combination with alcohol in moderate drinkers. *Psychopharmacology (Berl).* 2004;172(1):16–24. 10.1007/s00213-003-1617-5 14530901

[127] Krupitsky EM Neznanova O Masalov D: Effect of memantine on cue-induced alcohol craving in recovering alcohol-dependent patients. *Am J Psychiatry.* 2007;164(3):519–23. 10.1176/ajp.2007.164.3.519 17329479

[128] Schumann G Johann M Frank J: Systematic analysis of glutamatergic neurotransmission genes in alcohol dependence and adolescent risky drinking behavior. *Arch Gen Psychiatry.* 2008;65(7):826–38. 10.1001/archpsyc.65.7.826 18606955

[129] Biermann T Reulbach U Lenz B: *N*-methyl-D-aspartate 2b receptor subtype (NR2B) promoter methylation in patients during alcohol withdrawal. *J Neural Transm (Vienna).* 2009;116(5):615–22. 10.1007/s00702-009-0212-2 19350219

[130] Bach P Kirsch M Hoffmann S: The effects of single nucleotide polymorphisms in glutamatergic neurotransmission genes on neural response to alcohol cues and craving. *Addict Biol.* 2015;20(6):1022–32. 10.1111/adb.12291 26289945

[131] Wise RA: Voluntary ethanol intake in rats following exposure to ethanol on various schedules. *Psychopharmacologia.* 1973;29(3):203–10. 10.1007/BF00414034 4702273

[132] Simms JA Steensland P Medina B: Intermittent access to 20% ethanol induces high ethanol consumption in Long-Evans and Wistar rats. *Alcohol Clin Exp Res.* 2008;32(10):1816–23. 10.1111/j.1530-0277.2008.00753.x 18671810 PMC3151464

[133] Carnicella S Ron D Barak S: Intermittent ethanol access schedule in rats as a preclinical model of alcohol abuse. *Alcohol.* 2014;48(3):243–52. 10.1016/j.alcohol.2014.01.006 24721195 PMC4102254

[134] Hopf FW Chang SJ Sparta DR: Motivation for alcohol becomes resistant to quinine adulteration after 3 to 4 months of intermittent alcohol self-administration. *Alcohol Clin Exp Res.* 2010;34(9):1565–73. 10.1111/j.1530-0277.2010.01241.x 20586757 PMC2997761

[135] Hwa LS Chu A Levinson SA: Persistent escalation of alcohol drinking in C57BL/6J mice with intermittent access to 20% ethanol. *Alcohol Clin Exp Res.* 2011;35(11):1938–47. 10.1111/j.1530-0277.2011.01545.x 21631540 PMC3166538

[136] Hwa LS Nathanson AJ Shimamoto A: Aggression and increased glutamate in the mPFC during withdrawal from intermittent alcohol in outbred mice. *Psychopharmacology (Berl).* 2015;232(16):2889–902. 10.1007/s00213-015-3925-y 25899790 PMC4515187

[137] Hwa LS Debold JF Miczek KA: Alcohol in excess: CRF _1_ receptors in the rat and mouse VTA and DRN. *Psychopharmacology (Berl).* 2013;225(2):313–27. 10.1007/s00213-012-2820-z 22885872 PMC3518642

[138] McGuier NS Padula AE Lopez MF: Withdrawal from chronic intermittent alcohol exposure increases dendritic spine density in the lateral orbitofrontal cortex of mice. *Alcohol.* 2015;49(1):21–7. 10.1016/j.alcohol.2014.07.017 25468278 PMC4314373

[139] Stuber GD Hopf FW Hahn J: Voluntary ethanol intake enhances excitatory synaptic strength in the ventral tegmental area. *Alcohol Clin Exp Res.* 2008;32(10):1714–20. 10.1111/j.1530-0277.2008.00749.x 18627359 PMC3040033

[140] Cuzon Carlson VC Seabold GK Helms CM: Synaptic and morphological neuroadaptations in the putamen associated with long-term, relapsing alcohol drinking in primates. *Neuropsychopharmacology.* 2011;36(12):2513–28. 10.1038/npp.2011.140 21796110 PMC3194078

[141] Obara I Bell RL Goulding SP: Differential effects of chronic ethanol consumption and withdrawal on homer/glutamate receptor expression in subregions of the accumbens and amygdala of P rats. *Alcohol Clin Exp Res.* 2009;33(11):1924–34. 10.1111/j.1530-0277.2009.01030.x 19673743 PMC2873844

[142] Szumlinski KK Ary AW Lominac KD: Accumbens Homer2 overexpression facilitates alcohol-induced neuroplasticity in C57BL/6J mice. *Neuropsychopharmacology.* 2008;33(6):1365–78. 10.1038/sj.npp.1301473 17568396 PMC5099135

[143] Das SC Yamamoto BK Hristov AM: Ceftriaxone attenuates ethanol drinking and restores extracellular glutamate concentration through normalization of GLT-1 in nucleus accumbens of male alcohol-preferring rats. *Neuropharmacology.* 2015;97:67–74. 10.1016/j.neuropharm.2015.05.009 26002627 PMC4537362

[144] Rao PS Bell RL Engleman EA: Targeting glutamate uptake to treat alcohol use disorders. *Front Neurosci.* 2015;9:144. 10.3389/fnins.2015.00144 25954150 PMC4407613

[145] George O Sanders C Freiling J: Recruitment of medial prefrontal cortex neurons during alcohol withdrawal predicts cognitive impairment and excessive alcohol drinking. *Proc Natl Acad Sci U S A.* 2012;109(44):18156–61. 10.1073/pnas.1116523109 23071333 PMC3497825

[146] Seif T Chang SJ Simms JA: Cortical activation of accumbens hyperpolarization-active NMDARs mediates aversion-resistant alcohol intake. *Nat Neurosci.* 2013;16(8):1094–100. 10.1038/nn.3445 23817545 PMC3939030

[147] Griffin WC 3rd Lopez MF Becker HC: Intensity and duration of chronic ethanol exposure is critical for subsequent escalation of voluntary ethanol drinking in mice. *Alcohol Clin Exp Res.* 2009;33(11):1893–900. 10.1111/j.1530-0277.2009.01027.x 19673744 PMC2995298

[148] Griffin WC 3rd: Alcohol dependence and free-choice drinking in mice. *Alcohol.* 2014;48(3):287–93. 10.1016/j.alcohol.2013.11.006 24530006 PMC4032174

[149] Gilpin NW Smith AD Cole M: Operant behavior and alcohol levels in blood and brain of alcohol-dependent rats. *Alcohol Clin Exp Res.* 2009;33(12):2113–23. 10.1111/j.1530-0277.2009.01051.x 19740131 PMC2789881

[150] Rossetti ZL Carboni S: Ethanol withdrawal is associated with increased extracellular glutamate in the rat striatum. *Eur J Pharmacol.* 1995;283(1–3):177–83. 10.1016/0014-2999(95)00344-K 7498307

[151] Dahchour A De Witte P Bolo N: Central effects of acamprosate: part 1. Acamprosate blocks the glutamate increase in the nucleus accumbens microdialysate in ethanol withdrawn rats. *Psychiatry Res.* 1998;82(2):107–14. 10.1016/S0925-4927(98)00016-X 9754453

[152] Melendez RI Hicks MP Cagle SS: Ethanol exposure decreases glutamate uptake in the nucleus accumbens. *Alcohol Clin Exp Res.* 2005;29(3):326–33. 10.1097/01.ALC.0000156086.65665.4D 15770106

[153] Griffin WC 3rd Haun HL Hazelbaker CL: Increased extracellular glutamate in the nucleus accumbens promotes excessive ethanol drinking in ethanol dependent mice. *Neuropsychopharmacology.* 2014;39(3):707–17. 10.1038/npp.2013.256 24067300 PMC3895249

[154] Dahchour A De Witte P: Effect of repeated ethanol withdrawal on glutamate microdialysate in the hippocampus. *Alcohol Clin Exp Res.* 1999;23(10):1698–703. 10.1111/j.1530-0277.1999.tb04063.x 10550004

[155] Claus D Kim JS Kornhuber ME: [Effect of ethanol on the neurotransmitters glutamate and GABA]. *Arch Psychiatr Nervenkr (1970).* 1982;232(2):183–9. 10.1007/BF00343699 6130753

[156] Erden BF Ozdemirci S Yildiran G: Dextromethorphan attenuates ethanol withdrawal syndrome in rats. *Pharmacol Biochem Behav.* 1999;62(3):537–41. 10.1016/S0091-3057(98)00175-0 10080248

[157] Grant KA Valverius P Hudspith M: Ethanol withdrawal seizures and the NMDA receptor complex. *Eur J Pharmacol.* 1990;176(3):289–96. 10.1016/0014-2999(90)90022-X 2158451

[158] Stepanyan TD Farook JM Kowalski A: Alcohol withdrawal-induced hippocampal neurotoxicity *in vitro* and seizures *in vivo* are both reduced by memantine. *Alcohol Clin Exp Res.* 2008;32(12):2128–35. 10.1111/j.1530-0277.2008.00801.x 18828800

[159] Kroener S Mulholland PJ New NN: Chronic alcohol exposure alters behavioral and synaptic plasticity of the rodent prefrontal cortex. *PLoS One.* 2012;7(5):e37541. 10.1371/journal.pone.0037541 22666364 PMC3364267

[160] Radke AK Jury NJ Kocharian A: Chronic EtOH effects on putative measures of compulsive behavior in mice. *Addict Biol.* 2017;22(2):423–434. 10.1111/adb.12342 26687341 PMC4916036

[161] Meinhardt MW Hansson AC Perreau-Lenz S: Rescue of infralimbic mGluR _2_ deficit restores control over drug-seeking behavior in alcohol dependence. *J Neurosci.* 2013;33(7):2794–806. 10.1523/JNEUROSCI.4062-12.2013 23407939 PMC3711176

[162] Olive MF Becker HC: Effects of the mGluR2/3 agonist LY379268 and the mGluR5 antagonist MPEP on handling-induced convulsions during ethanol withdrawal in mice. *Alcohol.* 2008;42(3):191–7. 10.1016/j.alcohol.2008.01.007 18420113 PMC2386874

[163] Hu W Morris B Carrasco A: Effects of acamprosate on attentional set-shifting and cellular function in the prefrontal cortex of chronic alcohol-exposed mice. *Alcohol Clin Exp Res.* 2015;39(6):953–61. 10.1111/acer.12722 25903298 PMC10782929

[164] Abulseoud OA Camsari UM Ruby CL: Attenuation of ethanol withdrawal by ceftriaxone-induced upregulation of glutamate transporter EAAT2. *Neuropsychopharmacology.* 2014;39(7):1674–84. 10.1038/npp.2014.14 24452391 PMC4023140

[165] Lee MR Hinton DJ Wu J: Acamprosate reduces ethanol drinking behaviors and alters the metabolite profile in mice lacking ENT1. *Neurosci Lett.* 2011;490(2):90–5. 10.1016/j.neulet.2010.12.033 21172405 PMC3032037

[166] Nam HW Lee MR Zhu Y: Type 1 equilibrative nucleoside transporter regulates ethanol drinking through accumbal *N*-methyl-D-aspartate receptor signaling. *Biol Psychiatry.* 2011;69(11):1043–51. 10.1016/j.biopsych.2011.02.013 21489406 PMC3090461

[167] Sari Y: Potential therapeutic role of glutamate transporter 1 for the treatment of alcohol dependence. *OA Alcohol.* 2013;1(1):6. 10.13172/2053-0285-1-1-574 24409344 PMC3883353

[168] Griffin WC Ramachandra VS Knackstedt LA: Repeated cycles of chronic intermittent ethanol exposure increases basal glutamate in the nucleus accumbens of mice without affecting glutamate transport. *Front Pharmacol.* 2015;6:27. 10.3389/fphar.2015.00027 25755641 PMC4337330

[169] George O Koob GF: Individual differences in prefrontal cortex function and the transition from drug use to drug dependence. *Neurosci Biobehav Rev.* 2010;35(2):232–47. 10.1016/j.neubiorev.2010.05.002 20493211 PMC2955797

[170] Roberto M Schweitzer P Madamba SG: Acute and chronic ethanol alter glutamatergic transmission in rat central amygdala: an *in vitro* and *in vivo* analysis. *J Neurosci.* 2004;24(7):1594–603. 10.1523/JNEUROSCI.5077-03.2004 14973247 PMC6730456

[171] Freeman K Staehle MM Vadigepalli R: Coordinated dynamic gene expression changes in the central nucleus of the amygdala during alcohol withdrawal. *Alcohol Clin Exp Res.* 2013;37(Suppl 1):E88–100. 10.1111/j.1530-0277.2012.01910.x 22827539 PMC4408903

[172] Floyd DW Jung KY McCool BA: Chronic ethanol ingestion facilitates *N*-methyl-D-aspartate receptor function and expression in rat lateral/basolateral amygdala neurons. *J Pharmacol Exp Ther.* 2003;307(3):1020–9. 10.1124/jpet.103.057505 14534353

[173] Carpenter-Hyland EP Woodward JJ Chandler LJ: Chronic ethanol induces synaptic but not extrasynaptic targeting of NMDA receptors. *J Neurosci.* 2004;24(36):7859–68. 10.1523/JNEUROSCI.1902-04.2004 15356198 PMC6729936

[174] Kash TL Baucum AJ 2nd Conrad KL: Alcohol exposure alters NMDAR function in the bed nucleus of the stria terminalis. *Neuropsychopharmacology.* 2009;34(11):2420–9. 10.1038/npp.2009.69 19553918 PMC2864644

[175] Wills TA Klug JR Silberman Y: GluN2B subunit deletion reveals key role in acute and chronic ethanol sensitivity of glutamate synapses in bed nucleus of the stria terminalis. *Proc Natl Acad Sci U S A.* 2012;109(5):E278–87. 10.1073/pnas.1113820109 22219357 PMC3277158

[176] Masneuf S Lowery-Gionta E Colacicco G: Glutamatergic mechanisms associated with stress-induced amygdala excitability and anxiety-related behavior. *Neuropharmacology.* 2014;85:190–7. 10.1016/j.neuropharm.2014.04.015 24796255 PMC4170856

[177] Kallupi M Varodayan FP Oleata CS: Nociceptin/orphanin FQ decreases glutamate transmission and blocks ethanol-induced effects in the central amygdala of naive and ethanol-dependent rats. *Neuropsychopharmacology.* 2014;39(5):1081–92. 10.1038/npp.2013.308 24169802 PMC3957102

[178] Koob GF: A role for brain stress systems in addiction. *Neuron.* 2008;59(1):11–34. 10.1016/j.neuron.2008.06.012 18614026 PMC2748830

[179] Reynolds AR Berry JN Sharrett-Field L: Ethanol withdrawal is required to produce persisting N-methyl-D-aspartate receptor-dependent hippocampal cytotoxicity during chronic intermittent ethanol exposure. *Alcohol.* 2015;49(3):219–27. 10.1016/j.alcohol.2015.01.008 25746220 PMC4414743

[180] Brigman JL Wright T Talani G: Loss of GluN2B-containing NMDA receptors in CA1 hippocampus and cortex impairs long-term depression, reduces dendritic spine density, and disrupts learning. *J Neurosci.* 2010;30(13):4590–600. 10.1523/JNEUROSCI.0640-10.2010 20357110 PMC2869199

[181] Kiselycznyk C Svenningsson P Delpire E: Genetic, pharmacological and lesion analyses reveal a selective role for corticohippocampal GLUN2B in a novel repeated swim stress paradigm. *Neuroscience.* 2011;193:259–68. 10.1016/j.neuroscience.2011.06.015 21704131 PMC4796944

[182] Reissner KJ Kalivas PW: Using glutamate homeostasis as a target for treating addictive disorders. *Behav Pharmacol.* 2010;21(5–6):514–22. 10.1097/FBP.0b013e32833d41b2 20634691 PMC2932669

[183] McClearn GE Rodgers DA: Genetic factors in alcohol preference of laboratory mice. *J Comp Physiol Psychol.* 1961;54:116–9. 10.1037/h0042530

[184] Belknap JK Crabbe JC Young ER: Voluntary consumption of ethanol in 15 inbred mouse strains. *Psychopharmacology (Berl).* 1993;112(4):503–10. 10.1007/BF02244901 7871064

[185] Elmer GI Meisch RA George FR: Mouse strain differences in operant self-administration of ethanol. *Behav Genet.* 1987;17(5):439–51. 10.1007/BF01073111 3426501

[186] Melendez RI Rodd-Henricks ZA Engleman EA: Microdialysis of dopamine in the nucleus accumbens of alcohol-preferring (P) rats during anticipation and operant self-administration of ethanol. *Alcohol Clin Exp Res.* 2002;26(3):318–25. 10.1111/j.1530-0277.2002.tb02540.x 11923583

[187] Lê AD Quan B Juzytch W: Reinstatement of alcohol-seeking by priming injections of alcohol and exposure to stress in rats. *Psychopharmacology (Berl).* 1998;135(2):169–74. 10.1007/s002130050498 9497022

[188] Chaudhri N Sahuque LL Schairer WW: Separable roles of the nucleus accumbens core and shell in context- and cue-induced alcohol-seeking. *Neuropsychopharmacology.* 2010;35(3):783–91. 10.1038/npp.2009.187 19924113 PMC2813976

[189] Sinclair JD Walker S Jordan W: Behavioral and physiological changes associated with various durations of alcohol deprivation in rats. *Q J Stud Alcohol.* 1973;34(3):744–57. 4795453

[190] Melendez RI Middaugh LD Kalivas PW: Development of an alcohol deprivation and escalation effect in C57BL/6J mice. *Alcohol Clin Exp Res.* 2006;30(12):2017–25. 10.1111/j.1530-0277.2006.00248.x 17117967

[191] Goldstein DB: Relationship of alcohol dose to intensity of withdrawal signs in mice. *J Pharmacol Exp Ther.* 1972;180(2):203–15. 5062297

[192] O'Dell LE Roberts AJ Smith RT: Enhanced alcohol self-administration after intermittent versus continuous alcohol vapor exposure. *Alcohol Clin Exp Res.* 2004;28(11):1676–82. 10.1097/01.ALC.0000145781.11923.4E 15547454

[193] Lopez MF Becker HC: Effect of pattern and number of chronic ethanol exposures on subsequent voluntary ethanol intake in C57BL/6J mice. *Psychopharmacology (Berl).* 2005;181(4):688–96. 10.1007/s00213-005-0026-3 16001125

